# Effect of Zinc Oxide Nanoparticles on the Physiological and Biochemical Responses of *Coffea arabica* L. Exposed to Glyphosate

**DOI:** 10.3390/nano16010039

**Published:** 2025-12-27

**Authors:** Leyner Tucto-Vela, Jegnes Benjamín Meléndez-Mori, Eyner Huaman, Amilcar Valle-Lopez, Manuel Oliva Cruz

**Affiliations:** Instituto de Investigación para el Desarrollo Sustentable de Ceja de Selva (INDES-CES), Universidad Nacional Toribio Rodríguez de Mendoza (UNTRM), Chachapoyas 01001, Peru; leynertuctovela01@gmail.com (L.T.-V.);

**Keywords:** oxidative stress, nanoparticles, *Coffea*, glyphosate

## Abstract

Glyphosate is widely used for weed control in coffee but can induce physiological alterations due to its lack of selectivity, and indirect spray drift can cause adverse effects, potentially increasing biological impacts upon exposure. In this study, we evaluated the attenuating effect of foliar-applied zinc oxide nanoparticles (ZnO NPs) on *C. arabica* var. Geisha seedlings exposed to simulated spray concentrations of glyphosate (3.6 and 17.9 g ae L^−1^). Exposure caused a marked reduction in chlorophyll content, stomatal conductance, and net photosynthesis, while simultaneously promoting an increase in H_2_O_2_, MDA, and proline accumulation, reflecting a pronounced redox imbalance and oxidative damage associated with the production of reactive oxygen species (ROS). In contrast, the application of ZnO NPs improved photosynthetic efficiency, increased chlorophyll content, stabilized stomatal aperture, and reduced H_2_O_2_ and MDA levels in both leaves and roots. Moreover, it enhanced nutrient accumulation, ensuring greater membrane integrity and more efficient ion transport systems under glyphosate exposure. Overall, the ZnO NPs exhibited a notable protective effect by reducing glyphosate-induced phytotoxicity and strengthening the physiological tolerance of *C. arabica*. These findings support their potential as a sustainable tool to protect coffee crops from glyphosate exposure.

## 1. Introduction

Coffee (*Coffea arabica* L.) is recognized as a strategic crop for the economic development of producing countries and is, globally, among the most economically important agricultural export commodities [1]. It is estimated that, for the 2025/26 season, global coffee production will decrease by 500,000 bags from the December 2024 estimate, reaching 174.4 million bags as a consequence of drought conditions, elevated temperatures, and various biotic and abiotic factors affecting crop productivity [2,3]. Among these factors, weeds represent a major limitation by competing with coffee plants for light, water, nutrients, and root space, thereby reducing growth, weakening plant physiology, and decreasing yield [4]. In coffee production systems, weed management requires substantial labor, leading producers to rely extensively on herbicides [5,6]. Herbicide application is the most widely used method for chemical weed control due to its effectiveness and cost efficiency [7]. However, the indiscriminate use of these products and application errors can compromise both coffee production and quality, as herbicides may reach the crop directly or through spray drift, particularly in young plantations [8,9,10].

Glyphosate is one of the most widely used herbicides for weed control due to its high efficacy and broad spectrum of action, its low toxicity to animals, rapid degradation by microorganisms, and very short soil persistence [11]. It is a non-selective, broad-spectrum herbicide whose mode of action is based on the inhibition of the enzyme 5-enolpyruvylshikimate-3-phosphate synthase (EPSPS); once absorbed, it produces characteristic symptoms of damage and induces the accumulation of shikimic acid in plant tissues [12]. Owing to its limited selectivity, there is a risk that crops may be affected by accidental exposure during application, and it is estimated that up to 10% of the sprayed product can reach non-target plants, compromising their integrity and physiological performance, particularly in young plants [13,14]. Secondary effects of this herbicide have been reported on photosynthesis, leaf morphology, and secondary metabolites [15,16,17]. These physiological alterations are commonly associated with the overproduction of reactive oxygen species (ROS), leading to oxidative stress and cellular damage [18]. Therefore, herbicide selection is crucial, as it allows for effective weed control without harming the crop [11]. However, risk assessment in non-target plants requires species-specific information [19], and crop tolerance to these compounds is closely related to their ability to counteract the oxidative stress they induce [20].

The application of nanotechnology in agriculture has shown promising results in enhancing plant tolerance to various environmental factors [21], with plant responses largely depending on species-specific traits that govern nanoparticle absorption, accumulation, and translocation [22,23]. Within this framework, zinc-based nanomaterials have attracted particular interest due to their dual nutritional and physicochemical functions in plant systems. Among these nanomaterials, zinc oxide nanoparticles (ZnO NPs) have gained particular attention due to their high specific surface area, surface charge, and wide band-gap semiconductor nature, which enables efficient electron transfer reactions and photocatalytic activity, considering that surface charge can significantly modulate interactions with microorganisms [24,25]. In particular, green- or biosynthesized ZnO NPs have been reported to exhibit enhanced photocatalytic efficiency and broad-spectrum antimicrobial activity, attributes closely linked to their surface chemistry and redox behavior [26,27]. These physicochemical properties are biologically relevant, as surface-mediated redox reactions and controlled ROS generation associated with ZnO NPs photocatalytic reactivity directly influence nanoparticle–cell interactions and stress-related physiological responses in plants [28,29].

Despite their promising applications, ZnO NPs may pose environmental and phytotoxic risks when applied beyond safe concentration thresholds [30]. Reported effects include soil persistence, plant accumulation, and species-dependent sensitivity, underscoring the need for dose optimization and responsible use [31]. Furthermore, these nanomaterials need to be carefully evaluated for potential toxicity and to minimize their harmful effects on humans, animals, and plants [25].

In this context, experimental studies using ZnO NPs have demonstrated improved antioxidant capacity across several physiological processes and a reduction in reactive oxygen species generated under salt stress [32,33]. Likewise, they have been shown to increase photosynthetic pigments and total soluble protein content, as well as reduce MDA levels in *L. leucocephala* seedlings [34], in addition to lowering proline and H_2_O_2_ accumulation under stress conditions, thereby alleviating oxidative stress [35].

Therefore, considering the favorable results reported and the need to deepen the understanding of the physiological and biochemical activities influenced by ZnO NPs under stress conditions, the present study aimed to assess whether foliar application of ZnO NPs can mitigate glyphosate-induced physiological and oxidative damage in *C. arabica* seedlings. It was hypothesized that these nanoparticles can attenuate herbicide-induced damage by enhancing photosynthetic efficiency and strengthening antioxidant mechanisms, thereby reducing oxidative stress and preserving the physiological functionality of the plants.

## 2. Materials and Methods

### 2.1. Plant Material and Growth Conditions

Seedlings of *C. arabica* var. Geisha were used and maintained throughout the experiment in a greenhouse with 50% shade, under a natural photoperiod, at an average temperature of 20 °C and a relative humidity of 80%. Seedlings of homogeneous size and with suitable phytosanitary conditions were selected. The seedlings were planted in 1 kg pots. The substrate consisted of a mixture of agricultural soil, peat, and sand in a 4:2:2 ratio, respectively. The physical and chemical characteristics of the soils used in this experiment are presented in Supplementary Table S1 [36].

### 2.2. Preparation of Glyphosate Dilutions and ZnO NPs

Glyphosate (480 g L^−1^,technical grade; Sichuan Leshan Fuhua Tongda Agro-Chemical Technology Co., Ltd., Leshan, Sichuan, China), expressed as acid equivalent (ae), was used by applying a conversion factor of 0.747. Prior to the establishment of the experiment, preliminary dose–response assays were conducted to identify concentrations capable of inducing different levels of physiological damage in coffee seedlings. Based on these results, two doses were selected: a sublethal dose, prepared by diluting 10 mL of the commercial formulation to a final concentration of 3.6 g ae L^−1^, which induced measurable but moderate physiological alterations; and a stress-inducing dose, obtained from 50 mL of the formulation (17.9 g ae L^−1^), defined as the highest concentration that caused severe damage without compromising plant survival. Both solutions were diluted in 1 L of distilled water and applied via foliar spraying following the manufacturer’s recommendations. The stress dose exceeds typical spray-drift scenarios and should be interpreted as a worst-case exposure.

Zinc oxide nanoparticles (<100 nm particle size (TEM), ≤40 nm avg. part. size (APS), 20 wt.% in H_2_O, 7.5 ± 1.5 pH, density 1.7 g/mL ± 0.1 g/mL at 25 °C, purity > 99%; Sigma-Aldrich, St. Louis, MO, USA). Prior to application, ZnO NPs were dispersed in distilled water by sonication for 30 min. Nanoparticle suspensions were applied foliarly by a single spray to the dripping point (≈10 mL plant^−1^) in two stages: the first application was carried out three days after glyphosate application, and the second application was performed 30 days after the first ZnO-NP treatment. The experiment was maintained for 60 days, after which physiological and biochemical evaluations were conducted.

### 2.3. Experimental Setup and Design

The experiment comprised six treatments: (i) Control, (ii) ZnO (25 mg L^−1^), (iii) Glyphosate (3.6 g ae L^−1^), (iv) Glyphosate (3.6 g ae L^−1^) + ZnO (25 mg L^−1^), (v) Glyphosate (17.9 g ae L^−1^), and (vi) Glyphosate (17.9 g ae L^−1^) + ZnO (25 mg L^−1^). The experiment was arranged in a completely randomized design, with five plants per treatment.

Additionally, physiological and biochemical parameters were evaluated in each treatment to characterize the response of the coffee seedlings to the herbicide and the modulatory effect of ZnO NPs.

### 2.4. Net Photosynthetic Rate

Measurements were taken on fully expanded leaves from the middle third of C. arabica seedlings, 50 days after treatment initiation, using a LI-6800 portable photosynthesis system (LI-COR Biosciences, Lincoln, NE, USA). Evaluations were conducted between 10:00 and 11:00 h under the following chamber conditions: CO_2_ concentration of 400 nmol mol^−1^, airflow rate of 600 µmol s^−1^, photosynthetic photon flux density (PPFD) of 800 µmol m^−2^ s^−1^, relative humidity of 45%, leaf temperature of 21 °C, and fan speed of 2.5 rpm.

### 2.5. Determinaction of Relative Chlorophyll Content and Stomatal Conductance

The chlorophyll content index was measured using a SPAD-502 chlorophyll meter (Konica Minolta, Tokyo, Japan), while stomatal conductance (mmol m^−2^ s^−1^) was determined using a portable SC-1 foliar porometer (Decagon Devices, Pullman, WA, USA). Measurements were performed on all experimental plants, selecting two apical leaves per plant, and values were expressed as the mean of measurements recorded 30 days after stress onset.

### 2.6. Determination of Photosynthetic Pigments

The determination was carried out using 0.2 g of fresh leaves. The samples were macerated in a mortar over an ice bed using 0.2 g of magnesium carbonate and 6 mL of pre-chilled 80% acetone. The procedure was performed under indirect light in a Protector Basic 47 extractor hood (Labconco Corp., Kansas City, MO, USA). The resulting extract was transferred to amber Eppendorf tubes and centrifuged at 2800 rpm for 10 min at 12 °C. Subsequently, 2 mL of the supernatant were recovered, and absorbance readings were taken at 663.2, 646.8, and 470 nm in quartz cuvettes using a Genesys 180 UV/Vis spectrophotometer (Thermo Scientific™, Madison, WI, USA). The concentrations of photosynthetic pigments were calculated using the equations proposed by Lichtenthaler & Buschmann [37]. Results were expressed in µg g^−1^ fresh weight (FW):

$$Chlorophyll\;a(µg/\mathrm{mL})=12.25\;A_{663.2}-2.79\;A_{646.8}\;Chlorophyll\;b(µg/\mathrm{mL})=21.50\;A_{646.8}-5.10\;A_{663.2}\;Carotenoids\;(µg/\mathrm{mL})=(1000\;A_{470}-1.82\;Chl\;a-85.02\;Chl\;b)/198$$

### 2.7. Determination of Proline Content

To determine tissue proline content, the rapid colorimetric assay for proline quantification was performed [38]. A total of 500 mg of plant tissue was homogenized in 5 mL of 3% (*w*/*v*) aqueous sulfosalicylic acid using a horizontal shaker for 60 min, followed by centrifugation at 10,000 rpm for 30 min at 4 °C. Then, 1 mL of the supernatant was extracted and mixed with 1 mL of 0.1 M acidic ninhydrin and 1 mL of acetic acid. The reaction mixture was incubated at 90 °C for 60 min and subsequently cooled in an ice bath to stabilize the reaction. For the final extraction, 3 mL of toluene were added, and the absorbance of the upper phase was recorded at 520 nm using a Genesys 180 UV/Vis spectrophotometer (Thermo Scientific™, Madison, WI, USA), with toluene as the blank. Results were expressed in μg mg^−1^ fresh weight (FW).

### 2.8. Determination of Lipid Peroxidation

Lipid peroxidation was determined through the quantification of malondialdehyde (MDA) using the 2-thiobarbituric acid (TBA) assay, following the methodology with slight modifications [39]. For this purpose, 200 mg of fresh leaves or roots were homogenized in 2 mL of 0.1% (*w*/*v*) trichloroacetic acid (TCA). The resulting extract was centrifuged at 14,000 rpm for 10 min, and 1 mL of the supernatant was mixed with 2 mL of 0.5% TBA prepared in 20% TCA. The reaction mixture was incubated at 90 °C for 30 min and then rapidly cooled in an ice bath. Once the reaction was stabilized, the samples were centrifuged again at 14,000 rpm for 5 min. Absorbance readings of the supernatant were recorded at 450, 532, and 600 nm using a Genesys 180 UV/Vis spectrophotometer (Thermo Scientific™, Madison, WI, USA). MDA content was calculated using the formula of Wang et al. [40]:

$$MDA=\left(\mu molg^{-1}FW\right)=(6.45\times \left({OD}_{532}-{OD}_{600}\right)-0.56\times {OD}_{450}\times V/W$$

V = final volume of the extract (mL)

W = fresh weight of the sample (g)

### 2.9. Quantification of Hydrogen Peroxide Content 

The quantification of hydrogen peroxide (H_2_O_2_) was performed using a colorimetric assay based on potassium iodide. For this purpose, 200 mg of fresh tissue were homogenized in 2 mL of 0.1% trichloroacetic acid (TCA) and centrifuged at 12,000 rpm for 15 min. From the resulting supernatant, 1 mL was mixed with 1 mL of 10 mM phosphate buffer (pH 7.0) and 2 mL of 1 M potassium iodide (KI) [41]. The reaction mixture was incubated for 60 min at room temperature, and absorbance was subsequently measured at 390 nm using a Genesys 180 UV/Vis spectrophotometer (Thermo Scientific™, Madison, WI, USA).

The concentration of H_2_O_2_ was determined by interpolation from a standard curve constructed with dilutions of a 1 mM H_2_O_2_ stock solution, and the results were expressed in µmol g^−1^ fresh weight (FW).

### 2.10. Elemental Analysis

Dry samples (0.2 g) were digested in a nitric acid and chloride solution [42]. The solution was then filtered and the volume was adjusted to 25 mL with ultrapure water. The quantification of nutrients (P, K, Ca, Mg, Fe, Zn, Cu, Mn, Na) was performed using an Agilent 4100 microwave plasma–atomic emission spectrometer (MP-AES) (Agilent Technologies, Santa Clara, CA, USA). Results were expressed in µg g^−1^ of dry mass (DW) and calculated by interpolation from calibration curves obtained with standard solutions at different concentrations.

### 2.11. Stomatal Index and Density

Impressions of the abaxial leaf surface were taken at the point of maximum leaf width, near the midrib, using colorless nail polish and transparent cellophane tape [43]. Observations were made under an inverted optical microscope Leica DMi8 M (Leica Microsystems GmbH, Wetzlar, Germany). Observed within a field of view of 270.40 µm × 152.04 µm. Stomatal density (SD) was calculated as:

SD=Number of stomata/Area of the visual field (mm)

While the stomatal index (SI) was calculated as:

SI=[(stomata)/(total cells+stomata)]×100

### 2.12. Data Analysis

For variables that met the assumptions of normality (Shapiro–Wilk test; *p* > 0.05) and homogeneity of variances (Levene’s test; *p* > 0.05), a one-way analysis of variance (ANOVA) was performed. When significant effects were detected (*p* < 0.05), mean separation was conducted using the Tukey HSD test. In cases where these assumptions were not met, the non-parametric Kruskal–Wallis test was applied, followed by Dunn’s post hoc test for multiple comparisons when appropriate. All statistical analyses were conducted using R software (version 4.5.2) within the RStudio environment (version 2025.09.2+418).

## 3. Results

### 3.1. Relative Chlorophyll Content and Stomatal Conductance

**Figure 1:**
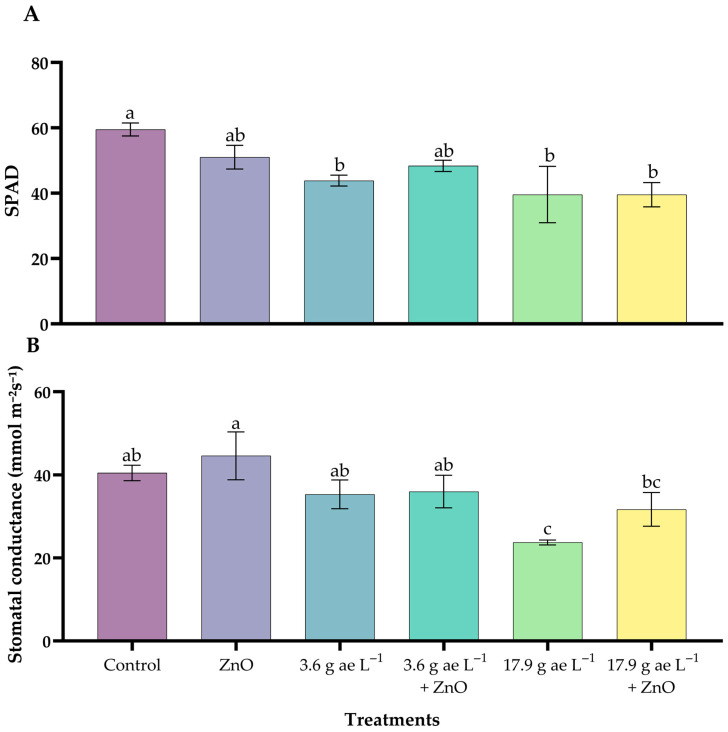
Effect of ZnO NPs on relative chlorophyll content (**A**) and stomatal conductance (**B**) in *C. arabica* seedlings under glyphosate exposure. Different letters above the bars indicate significant differences among treatments (*p* ≤ 0.05).

Seedlings treated with glyphosate alone showed significantly lower SPAD values compared with the control, whereas the combined application of glyphosate and ZnO NPs resulted in higher SPAD values than glyphosate-only treatments, approaching those of the control group (Figure 1A).

Regarding stomatal conductance (Figure 1B), the highest glyphosate dose (17.9 g ae L^−1^) resulted in the lowest conductance values, while plants receiving ZnO NPs exhibited higher stomatal conductance compared with their respective glyphosate-only treatments. The ZnO-only treatment displayed the highest stomatal conductance among all treatments. Zinc oxide nanoparticles in both variables strengthened tolerance capacity and mitigated the inhibitory effects of glyphosate.

### 3.2. Net Photosynthetic Rate (µmol CO_2_ m^−2^ s^−1^)

**Figure 2:**
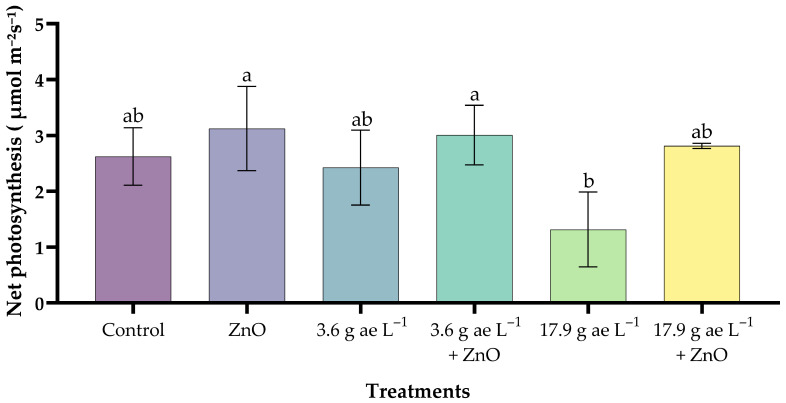
Effects of ZnO NPs on the net photosynthetic rate of coffee seedlings in the presence/absence of glyphosate. Different letters above the bars indicate significant differences (*p* ≤ 0.05).

Plants treated with zinc oxide nanoparticles (25 mg L^−1^) showed the highest value (3.12 ± 0.75 µmol CO_2_ m^−2^ s^−1^), exceeding the control (2.62 ± 0.52 µmol CO_2_ m^−2^ s^−1^) by 19%. The application of 17.9 g ae L^−1^ glyphosate (1.32 ± 0.67 µmol CO_2_ m^−2^ s^−1^) caused a significant reduction in photosynthesis (Figure 2). In contrast, seedlings exposed to 3.6 g ae L^−1^ glyphosate and treated with ZnO NPs maintained the highest photosynthetic rates, demonstrating a mitigating effect of the nanoparticles against the herbicide. Finally, at 17.9 g ae L^−1^ glyphosate, the ZnO-treated seedlings (2.81 ± 0.05 µmol CO_2_ m^−2^ s^−1^) showed partial recovery, confirming the protective effect of ZnO under glyphosate-induced stress conditions.

### 3.3. Photosynthetic Pigments Content

**Figure 3:**
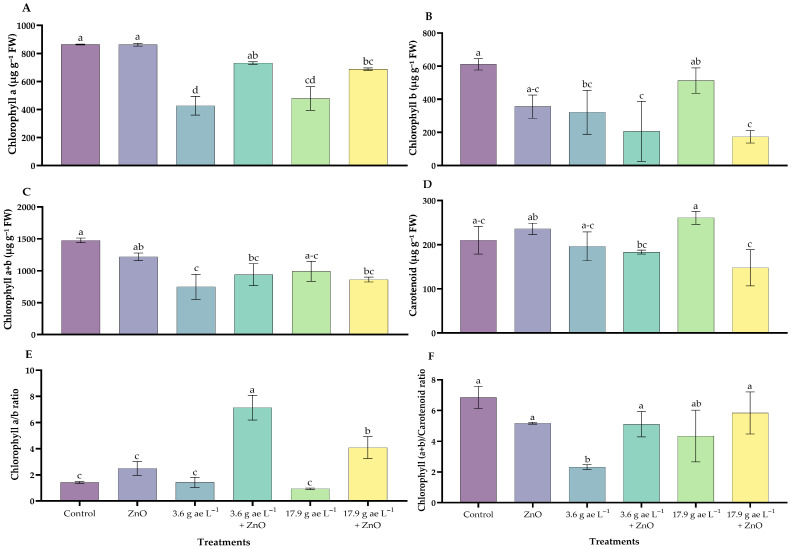
Photosynthetic pigment content in *C. arabica* under treatments with zinc oxide nanoparticles in the presence/absence of glyphosate. (**A**) Chlorophyll a, (**B**) Chlorophyll b, (**C**) Total chlorophyll (a + b), (**D**) Carotenoids, (**E**) Chlorophyll a/b ratio, and (**F**) Chlorophyll (a + b)/Carotenoids ratio. Different letters above the bars indicate significant differences (*p* ≤ 0.05).

The photosynthetic pigment content in *C. arabica* treated with ZnO NPs in the presence or absence of glyphosate is presented in Figure 3. Chlorophyll a content showed significant differences among treatments, with high values in seedlings treated with ZnO NPs (862.9 ± 5.81 µg g^−1^ FW) and in the control (864.50 ± 1.62 µg g^−1^ FW), whereas seedlings exposed to 3.6 g ae L^−1^ glyphosate (427.09 ± 38.43 µg g^−1^ FW) exhibited the lowest levels, representing a 51% reduction compared with ZnO NP–treated seedlings (Figure 3A).

Chlorophyll b content also differed significantly among treatments. The lowest concentration was recorded in seedlings exposed to 3.6 g ae L^−1^ glyphosate combined with ZnO NPs (173.49 ± 38.10 µg g^−1^ FW), whereas the highest value was observed in the control treatment (610.82 ± 33.81 µg g^−1^ FW), which was 72% higher than that recorded in the ZnO 25 mg L^−1^ treatment (Figure 3B).

For both pigments, ZnO NP–treated seedlings showed higher chlorophyll contents compared with those exposed to glyphosate alone. Total chlorophyll followed a similar trend, with significant reductions observed under glyphosate treatments and partial recovery in the presence of ZnO NPs (Figure 3C).

Regarding carotenoids, the highest glyphosate dose (17.9 g ae L^−1^) resulted in significantly higher carotenoid levels (260.81 ± 14.65 µg g^−1^ FW) compared with the control and ZnO NP–treated plants. When ZnO NPs were combined with glyphosate, carotenoid contents were lower than in the corresponding glyphosate-only treatments, particularly at the lower herbicide dose (Figure 3D). Chlorophyll a/b and (chlorophyll a + b)/carotenoids ratios also differed significantly among treatments, indicating changes in pigment composition associated with glyphosate exposure and ZnO NPs application (Figure 3E,F).

### 3.4. Proline Content

**Figure 4:**
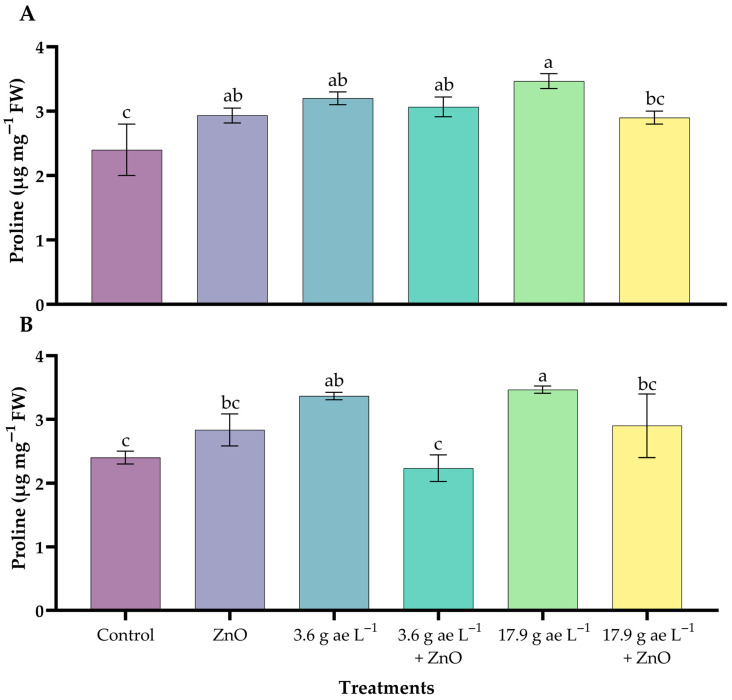
Proline content in coffee seedlings treated with nanoparticles in the presence/absence of glyphosate. (**A**) leaves and (**B**) roots. Different letters above the bars indicate significant differences (*p* ≤ 0.05).

Significant differences were observed among treatments in both leaves and roots (*p* < 0.05). Proline accumulation increased significantly in the leaves (Figure 4A), showing the highest values under 17.9 g ae L^−1^ glyphosate (3.44 ± 0.12 µg mg^−1^ FW). Likewise, 3.6 g ae L^−1^ glyphosate (3.20 ± 0.08 µg mg^−1^ FW) also increased proline. In root tissue, proline showed a similar response to glyphosate, with a slight increase (Figure 4B). The application of ZnO NPs at both glyphosate doses in leaves and roots partially reduced proline accumulation.

### 3.5. Lipid Peroxidation

**Figure 5:**
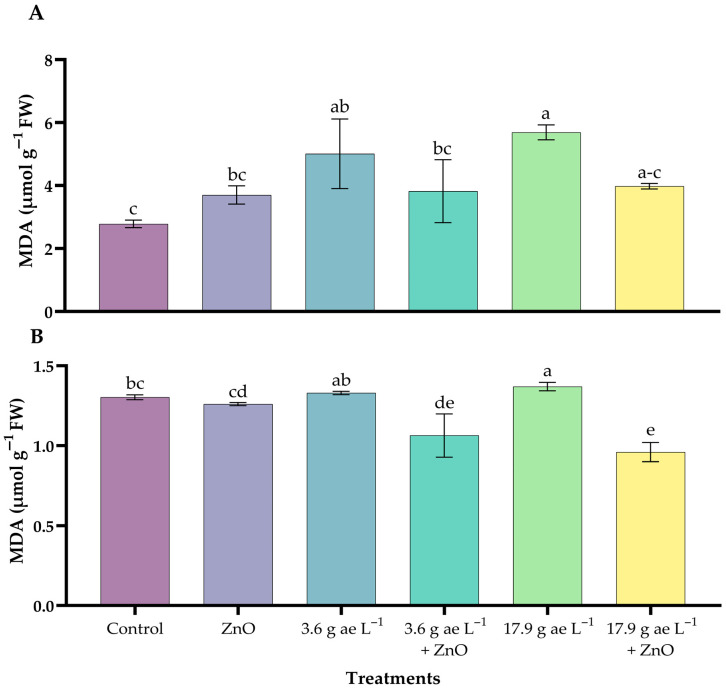
MDA content in coffee seedlings treated with ZnO NPs in the presence/absence of glyphosate. (**A**) leaves and (**B**) roots. Different letters above the bars indicate significant differences (*p* ≤ 0.05).

Lipid peroxidation increased with the application of glyphosate, with the highest values observed at the stress dose in both leaves and roots. In leaves (Figure 5A), the glyphosate dose of 17.9 g ae L^−1^ (5.69 ± 0.23 µmol g^−1^ FW) increased MDA levels compared with the control and ZnO nanoparticle-treated seedlings, whereas in roots the increase was more moderate at both glyphosate doses (Figure 5B). The incorporation of zinc oxide nanoparticles (ZnO-NPs) reduced malondialdehyde (MDA) levels in both tissues of glyphosate-exposed seedlings.

### 3.6. Hydrogen Peroxide (H_2_O_2_)

**Figure 6:**
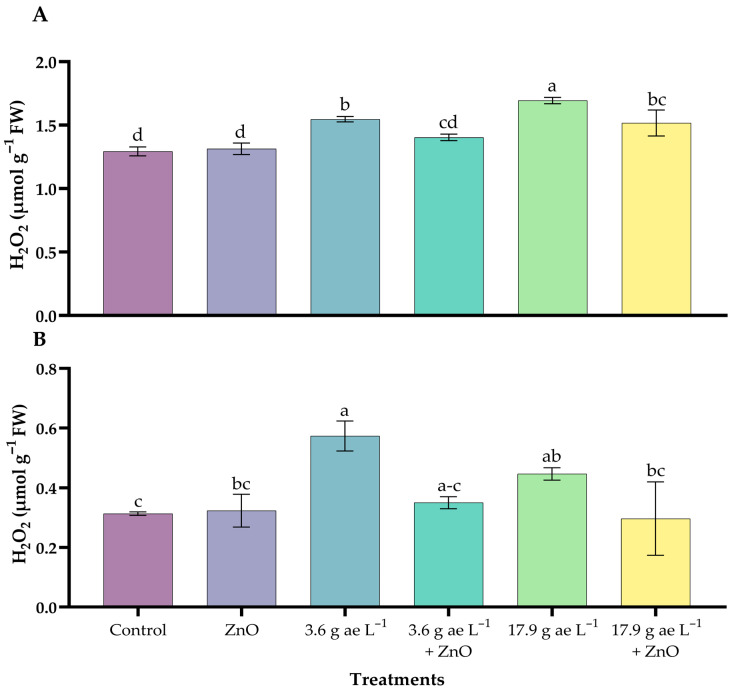
H_2_O_2_ content in coffee seedlings treated with nanoparticles in the presence/absence of glyphosate. (**A**) Leaves and (**B**) roots. Different letters above the bars indicate significant differences (*p* ≤ 0.05).

H_2_O_2_ concentrations increased with the application of glyphosate, showing significant differences compared to the other treatments. In the leaves, 17.9 g ae L^−1^ of glyphosate (1.69 ± 0.03 µmol g^−1^ FW) increased peroxide levels compared to the other treatments, which ranged from 1.29 to 1.55 units (Figure 6A).

On the other hand, in root tissues, seedlings exposed to 3.6 g ae L^−1^ of glyphosate (0.57 ± 0.03 µmol g^−1^ FW) showed the highest levels of hydrogen peroxide; however, the presence of ZnO NPs reduced these levels in both glyphosate doses (Figure 6B).

### 3.7. Nutrients Content

In Table 1, the nutrient content in the leaves of *C. arabica* under ZnO nanoparticle treatments in the presence/absence of glyphosate is shown. Seedlings treated with ZnO NPs presented the highest values for nutrients such as P (1410.1 ± 42.5 µg g^−1^ DW), Ca (16,203.9 ± 838.1 µg g^−1^ DW), Na (5191.8 ± 245.5 µg g^−1^ DW), K (13,747.6 ± 882.3 µg g^−1^ DW), Mn (49.9 ± 3.4 µg g^−1^ DW), and Zn (11.01 ± 0.8 µg g^−1^ DW). Meanwhile, some nutrients showed increases in seedlings treated with 3.6 g ae L^−1^ glyphosate plus ZnO NPs, such as Fe (104.9 ± 1.12 µg g^−1^ DW), Mg (2600.1 ± 295.2 µg g^−1^ DW), and Zn (11.7 ± 1.04 µg g^−1^ DW).

On the other hand, at 17.9 g ae L^−1^ glyphosate, nutrient levels remained mostly low. This occurred because at low doses, nanoparticles can maintain membrane integrity and mineral transport activity, reduce oxidative damage and favor nutrient translocation to the aerial parts. In contrast, at high concentrations, stress is more severe and partially limits the plant’s capacity to mobilize nutrients, even when the nanoparticles exert a mitigating effect.

In Table 2, root tissues show greater nutrient accumulation compared to leaves, as they act as the primary site of absorption, storage, and regulation under abiotic stress conditions. Treatments with ZnO NPs increased certain nutrients, such as P (2114.6 ± 41.8 µg g^−1^ DW), Fe (784.9 ± 8.9 µg g^−1^ DW), and Mg (4336.6 ± 78.8 µg g^−1^ DW).

In both glyphosate doses, most nutrient levels were low compared with the control; however, when treated with ZnO NPs, root nutritional content improved. This behavior is attributed to the nanoparticles enhancing membrane integrity, maintaining ion transporter activity, and reducing oxidative damage, which normally limits mineral uptake under glyphosate exposure.

### 3.8. Foliar Stomatal Structure

**Figure 7:**
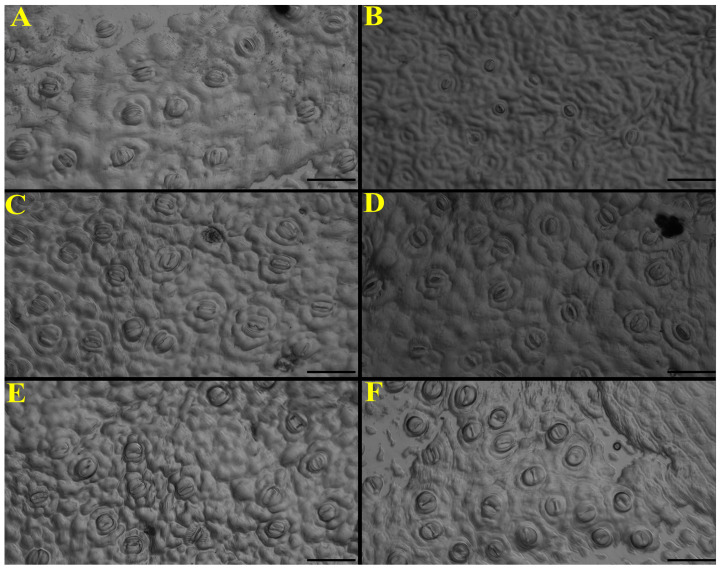
Images of stomatal structure under nanoparticle treatments in the presence/absence of glyphosate. (**A**) Control; (**B**) Glyphosate (17.9 g ae L^−1^); (**C**) Glyphosate (3.6 g ae L^−1^); (**D**) Glyphosate (17.9 g ae L^−1^) + ZnO (25 mg L^−1^); (**E**) Glyphosate (3.6 g ae L^−1^) + ZnO (25 mg L^−1^); (**F**) ZnO 25 mg L^−1^. Black scale bar = 50 µm.

Inverted microscopy (20×) revealed clear treatment-dependent differences in stomatal morphology (Figure 7): control plants exhibited regular and well-defined stomata (Figure 7A), whereas the stress dose markedly reduced stomatal aperture and sharpness (Figure 7B); the sublethal dose induced minimal and irregular openings (Figure 7C). In contrast, the application of ZnO NPs in stressed plants preserved visible and better-defined stomata, particularly under sublethal stress (Figure 7D,E), while seedlings treated exclusively with ZnO NPs showed abundant, well-distributed stomata with clearly defined contours (Figure 7F).

**Figure 8:**
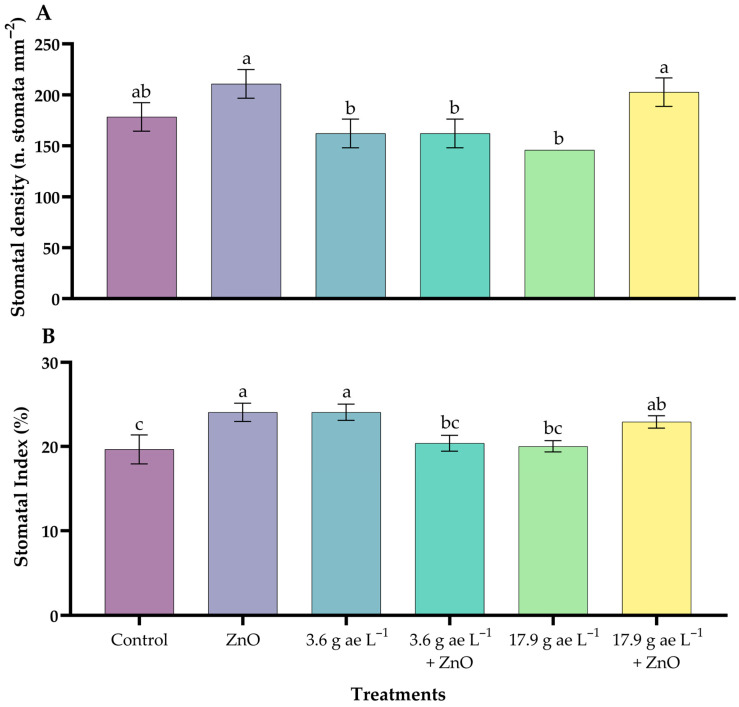
Stomatal density (**A**) and stomatal index (**B**) in the leaves of *C. arabica*. Different letters above the bars indicate significant differences (*p* ≤ 0.05).

Significant differences were observed between treatments with a greater tendency in stomatal density (SD) and stomatal index (SI) in coffee seedlings with ZnO NPs (Figure 8). The seedlings treated with ZnO NPs (210.77 ± 8.11 n. stomata mm^−2^) had the highest stomatal density compared to the other treatments, showing an approximate increase of 18% compared to the control (178.33 ± 8.11). Likewise, the stomatal index of the seedlings treated with ZnO NPs (24.06 ± 1.10) was significantly higher compared to the control (19.65 ± 1.72). In addition, the application of ZnO NPs improved the treatments in seedlings exposed to doses of glyphosate, attenuating the negative effects of the herbicide.

### 3.9. Correlation Analysis

**Figure 9:**
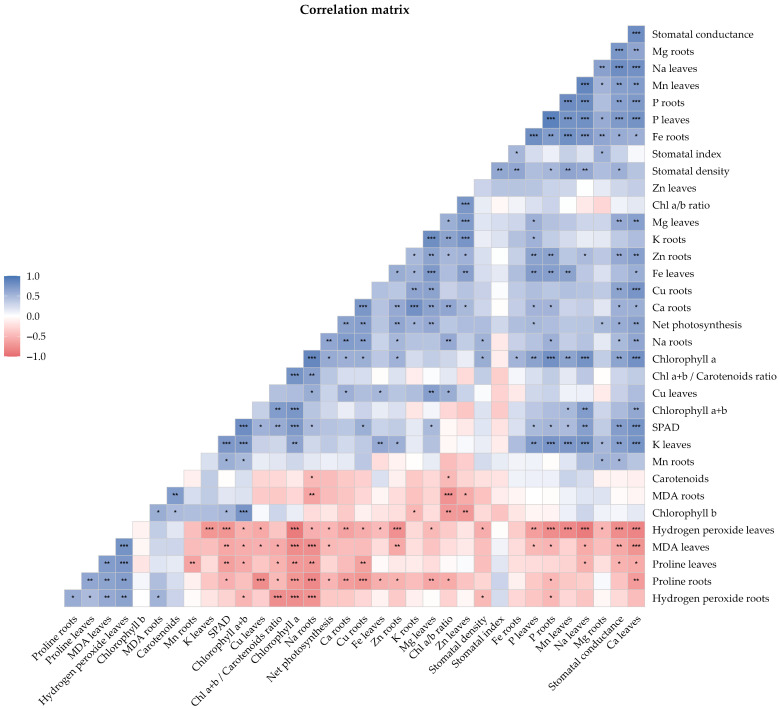
Correlation matrix of physiological, biochemical, and mineral parameters. The lower triangle shows correlation coefficients, with asterisks indicating statistical significance (*, *p* < 0.05; **, *p* < 0.01; ***, *p* < 0.001). Pearson’s or Spearman’s correlation coefficients were applied according to data normality.

Correlation analysis revealed multiple significant associations between the physiological, nutritional, and biochemical parameters evaluated (Figure 9). Among the strongest correlations (*p* < 0.001), phosphorus content showed a high degree of coordination between plant organs, with a strong positive correlation between leaves and roots (r = 0.896; *p* = 4.79 × 10^−7^). Oxidative stress in the leaves, represented by hydrogen peroxide (H_2_O_2_) content, was strongly associated with a reduction in photosynthetic pigments, as indicated by a negative correlation with chlorophyll a (r = −0.892; *p* = 6.55 × 10^−7^), as well as with decreased sodium content in leaves (r = −0.891; *p* = 7.05 × 10^−7^). Furthermore, foliar calcium emerged as a central component within the correlation network, showing positive associations with key indicators of photosynthetic function, including stomatal conductance (r = 0.815; *p* = 3.86 × 10^−5^), SPAD index (r = 0.755; *p* = 2.91 × 10^−4^), and chlorophyll a content (r = 0.778; *p* = 1.44 × 10^−4^).

In addition, a marked antagonistic relationship was observed between proline accumulation in roots and sodium content in roots (r = −0.815; *p* = 3.80 × 10^−5^), suggesting an interaction between osmoprotective responses and ionic balance. Membrane damage, assessed by MDA content, was positively associated with H_2_O_2_ (leaves: r = 0.768; *p* = 1.96 × 10^−4^) and negatively correlated with photosynthetic pigments (chlorophyll a: r = −0.784; *p* = 1.19 × 10^−4^), reinforcing the consistency of these biomarkers as indicators of plant stress status under the experimental conditions.

## 4. Discussion

Zinc oxide nanoparticles have emerged as a valuable tool in agronomic applications [44]. Their application enhances photosynthetic efficiency by supplying bioavailable zinc (Zn), a crucial cofactor required for optimal chloroplast function and the catalytic activity of various enzymes [45]. It was observed that glyphosate reduced net photosynthesis levels, a result that aligns with other studies reporting that this herbicide inhibits 5-enolpyruvylshikimate-3-phosphate synthase (EPSPS), an essential enzyme of the shikimate pathway through which approximately 20% or more of fixed carbon is transported [46]. Importantly, the application of ZnO NPs mitigated this inhibitory effect, improving net photosynthesis in glyphosate-treated seedlings. This finding is consistent with the research of Faizan et al. [47] and Sun et al. [45], who reported that zinc oxide nanoparticles promoted a slight increase in net photosynthetic rate, enhancing photosynthetic efficiency and stimulating antioxidant enzyme systems.

The presence of glyphosate reduced the relative chlorophyll content and stomatal conductance, in agreement with previous studies showing that this herbicide inhibits chlorophyll accumulation, disrupts thylakoid integrity, and impairs PSII function by interfering with the shikimate pathway and increasing oxidative stress [48,49]. This reduction also reflects stomatal closure as a defensive response to glyphosate exposure [50]. In the present study, ZnO NPs effectively enhanced physiological resilience under oxidative stress, improving both chlorophyll index and stomatal conductance. This protective effect is attributed to the gradual release of Zn^2+^, a key cofactor of superoxide dismutase (SOD), which enhances antioxidant defenses and reduces ROS-induced damage [51,52]. Moreover, Zn acts as a cofactor for carbonic anhydrase (CA), facilitating more efficient CO_2_ assimilation, as previously reported in maize and chickpea [53], and recent evidence suggests that specific NP doses can enhance metabolite production and alleviate nutritional deficiencies [54].

Likewise, the application of ZnO NPs had an effect on stomatal behavior. The decrease in stomatal density and index observed with glyphosate reflects its phytotoxic effect on stomatal formation and functionality, associated with oxidative stress and inhibition of shikimic acid metabolism [55]. However, the joint application with ZnO NPs mitigated these effects, showing higher stomatal density and index in seedlings treated with ZnO NPs, which is consistent with the findings of Ahmed et al. [56], who attribute this effect to the stimulation of cell division and regulation.

Photosynthetic pigments were analyzed to gain a more comprehensive understanding of the effects of ZnO NPs on coffee physiology. The results indicated that coffee plants exposed to glyphosate exhibited a significant decrease in chlorophyll *a* and chlorophyll *b* content, which is consistent with studies reporting alterations in the photosynthetic capacity for energy conversion in the reaction centers of photosystem II (PSII), a highly sensitive bioindicator of chemical stress [57,58]. Application of ZnO NPs restored chlorophyll levels, highlighting the role of zinc in chlorophyll biosynthesis via protochlorophyllide and in chloroplast development [59]. This behavior aligns with findings in other crops, where nanoparticles penetrate the leaf epidermis through stomata, accumulate in plastids, and translocate to chloroplasts [60].

Total chlorophyll followed a similar trend, with glyphosate reducing pigment content while ZnO NPs promoted partial recovery, likely due to activation of antioxidant defense systems that mitigate oxidative damage [61]. In this context, carotenoids also play a central role, functioning as protective pigments that safeguard plants from biotic and abiotic stress [33,62]. Their predominance under high glyphosate doses (Figure 3D) is consistent with other reports, where elevated H_2_O_2_ levels increase ROS concentrations and trigger carotenoid-mediated protective responses that prevent PSII damage [63,64].

Foliar application of ZnO NPs in glyphosate-exposed seedlings increased the *chlorophyll a/b ratio*, reflecting a higher relative concentration of PSI compared with PSII. This shift suggests an adjustment in light-harvesting complexes and thylakoid organization, consistent with previous reports in lettuce [65,66]. Similarly, the *Chlorophyll (a + b)/Carotenoids ratio* increased, indicating activation of photoprotective mechanisms that preserve PSII structure and enhance Rubisco efficiency [67,68].

Our study demonstrated increased malondialdehyde (MDA) levels in foliar and root tissues under glyphosate exposure (Figure 5A,B), confirming the induction of oxidative stress in *Coffea arabica*. Foliar tissues were more strongly affected, likely due to direct exposure to glyphosate. This response is consistent with previous reports identifying MDA as a key biomarker of oxidative damage in plants exposed to glyphosate and paraquat [69,70,71]. The application of ZnO NPs reduced MDA accumulation in glyphosate-treated plants, supporting their role in attenuating oxidative damage [72].

Hydrogen peroxide (H_2_O_2_) accumulation further confirmed glyphosate-induced oxidative stress, while proline accumulation reflected an adaptive antioxidant and osmoprotective response. Proline is known to contribute to ROS detoxification, NADP^+^ regeneration, and stress tolerance, and its accumulation under glyphosate exposure has been previously reported [73,74]. In this study, ZnO NP application enhanced this protective response, mitigating free radical–induced damage under excessive ROS and singlet oxygen production, in agreement with earlier findings [75].

Coffee seedlings treated with ZnO NPs showed greater foliar and root accumulation of P compared with control plants, with slight improvements also observed when nanoparticles were applied together with glyphosate. This increase promotes chlorophyll synthesis and photosynthesis, enhancing tolerance to abiotic stress factors [76]. Similarly, Burman et al. [77] demonstrated improvements in net photosynthesis, leaf area, chlorophyll content, and nitrogen metabolism. Potassium (K^+^) levels were also higher, stimulating its accumulation in mesophyll cells and improving photosynthetic efficiency [78]. In root tissue, ZnO NPs minimized stress effects through ionic balance and osmotic adjustment [79].

Multiple studies have reported increased Zn^2+^ accumulation in both leaves and roots compared with control treatments, a pattern also observed in the present study. Prasad et al. [80], showed that rice roots absorb more Zn when exposed to higher doses of ZnO NPs, a behavior that was likewise evident in our experiment. Regarding Ca^2+^ levels, ZnO NPs influenced calcium accumulation in both leaves and roots, particularly under glyphosate exposure, consistent with the role of Ca^2+^ in stress signaling and antioxidant regulation [35,81]. Moreover, the increase in Ca^2+^ concentration may be partially attributed to the ZnO nanoparticle–induced overexpression of genes associated with cation/H^+^ antiporter 18-like, calcium-transporting ATPase 13, and autoinhibited Ca^2+^ transporting ATPase 10 [82].

For leaf Cu^2+^ levels, a significant increase was observed in seedlings exposed to low-dose glyphosate and treated with ZnO NPs, activating their defense system and increasing micronutrient demand. This response aligns with Xu et al. [83], who demonstrated that copper functions as an antioxidant that suppresses oxidative stress.

Nanoparticles are known to increase mineral concentrations in leaves and roots [73]. In our study, most nutrients followed this pattern, including Mg, Mn, and Fe. This increase may be attributed to the ability of ZnO NPs to enhance the production of organic acids exuded by maize roots or to stimulate aquaporins through the overexpression of the *Tip1:1* and *Pip1:1* genes, which improves water and nutrient uptake in barley cells [84,85].

Several scientific studies indicate that nanoparticles can generate either beneficial or harmful effects in plants, depending primarily on their size, exposure time, dose, and the plant species involved [86,87]. In this context, nanoparticles can help correct nutritional deficiencies, increase stress resistance, and optimize both yield and crop quality, which are determined by their physicochemical properties and the surrounding environmental conditions [88,89]. However, inappropriate nanoparticle concentrations may cause toxicity by disrupting homeostasis, nutrient absorption, or interactions among different elements [90]. According to Bhatt et al. [91], nanoparticles can reduce lipid peroxidation and increase photosynthetic pigments, thereby contributing to plant stress tolerance through stabilization of reactive oxygen species (ROS) production. Nevertheless, they can also generate ROS capable of inducing controlled oxidative damage in microorganisms, whereas, at moderate concentrations, they activate endogenous antioxidant pathways, promoting the restoration of cellular homeostasis, and their interaction with biological membranes is associated with ionic release and alterations in membrane potential [25].

Several studies have demonstrated that ZnO NPs influence plant growth and yield, as well as Zn accumulation in different plant tissues [92]. ZnO NPs have been shown to promote plant growth up to a specific concentration threshold [47]. In this regard, concentrations between 25 and 50 mg L^−1^ have been associated with improvements in quality and yield parameters across various crops, along with a reduction in oxidative stress [93,94]. Nevertheless, it has also been reported that even at low concentrations, these nanoparticles may affect cellular processes such as cell division and genomic stability, highlighting the need for species-specific risk assessments and optimization of application rates to balance mitigation benefits with ecological safety [95].

These nanoparticles represent a promising alternative for addressing critical agricultural challenges such as climate change, soil and water degradation, and yield reductions caused by stress. Moreover, their study highlights the development of sustainable solutions and resilient cropping systems, underscoring their potential impact on modern agriculture.

## 5. Conclusions

Zinc oxide nanoparticles represent an alternative capable of mitigating the phytotoxic effects of glyphosate in *C. arabica* under controlled conditions. Their application improves photosynthetic efficiency, helps maintain the integrity of the pigment system, stabilizes stomatal dynamics, and reinforces antioxidant mechanisms against oxidative stress. The recovery of chlorophyll levels, adjustments in carotenoids, reduction in MDA, and improvement in the availability of essential nutrients support their protective role and their contribution to the physiological resilience of seedlings exposed to herbicides at an early stage of development. In this sense, the changes observed in stomatal density and stomatal index provide evidence that the application of ZnO NPs contributes to improved stomatal functionality under glyphosate-induced stress. Likewise, their positive effects on ionic homeostasis, redox balance, and PSII stability demonstrate that these nanoparticles not only reduce glyphosate-associated damage, but also optimize metabolic processes related to growth and stress tolerance. Under this approach, nanoparticles emerge as a promising tool at the experimental scale to improve agricultural sustainability and productivity, provided that they are applied at appropriate concentrations that optimize their benefits and minimize environmental risks.

## Figures and Tables

**Table 1:** Nutrient content (µg g^−1^ DW) in leaves of *C. arabica* treated with ZnO NPs in the presence/absence of glyphosate.

Treatment	P	Ca	Na	K	Fe	Mg	Zn	Cu	Mn
Control	777.6 ± 72.03 bc	15,632.8 ± 744.4 ab	3678.9 ± 374.3 ab	12,890.5 ± 679.5 ab	71.5 ± 10.9 bc	1995.5 ± 126.2 bc	3.12 ± 1.1 c	11.9 ± 1.5 a	34.9 ± 1.9 ab
ZnO 25 mg L^−1^	1410.1 ± 42.5 a	16,203.9 ± 838.9 a	5191.8 ± 245.5 a	13,747.6 ± 882.3 a	101.3 ± 18.9 ab	2195.8 ± 151.1 ab	11.01 ± 0.8 a	7.1 ± 0.8 ab	49.9 ± 3.4 a
Glyphosate (3.6 g ae L^−1^)	607.3 ± 97.8 cd	13,940.5 ± 852.1 bc	1272.7 ± 73.7 cd	10,569.1 ± 1182 bc	71.8 ± 6.6 bc	2181.2 ± 168.9 abc	7.8 ± 0.7 b	6.5 ± 1.1 b	28.7 ± 1.3 b
Glyphosate (3.6 g ae L^−1^) + ZnO 25	937.9 ± 48.8 b	14,862.7 ± 780.8 abc	1483.9 ± 77.4 bc	12,152.9 ± 985.2 ab	104.9 ± 1.12 a	2600.1 ± 295.2 a	8.93 ± 0.9 ab	14.4 ± 1.9 a	32.4 ± 2.5 ab
Glyphosate (17.9 g ae L^−1^)	464.6 ± 58.50 d	11,145.8 ± 427.9 d	841.3 ± 87.7 d	10,873.6 ± 573.6 bc	66.6 ± 5.5 c	1359.9 ± 143.1 d	3.1 ± 1.2 c	5.6 ± 0.1 b	27.7 ± 0.6 b
Glyphosate (17.9 g ae L^−1^) + ZnO 25	527.3 ± 213.3 cd	12,800.4 ± 971 cd	1302 ± 145.9 cd	9111.1 ± 924.8 c	59.8 ± 15.3 c	1704.7 ± 84.6 cd	7.11± 1.4 b	6.9 ± 0.5 ab	28.7 ± 3.4 b
CV (%)	13.50	5.61	-	7.73	14.41	8.68	15.28	-	-
*p*-value	1.39 × 10^−6^	4.41 × 10^−5^	0.0091	0.00041	0.0014	3 × 10^−5^	3.3 × 10^−6^	0.018	0.31

Values correspond to the mean ± standard deviation. Different letters within the same column indicate significant differences according to the statistical test performed.

**Table 2:** Nutrient content (µg g^−1^ DW) in roots of *C. arabica* treated with ZnO NPs in the presence/absence of glyphosate.

Treatment	P	Ca	Na	K	Fe	Mg	Zn	Cu	Mn
Control	1712.1 ± 74.1 bc	12,322.6 ± 255.1 a	4395.3 ± 136.2 a	14,182.3 ± 866.9 bc	435.3 ± 54.8 ab	4080.4 ± 89.3 abc	20.6 ± 5.5 ab	19.2 ± 2.2 a	56.1 ± 1.2 a
ZnO 25 mg L^−1^	2114.6 ± 41.8 a	12,355.6 ± 1442.2 a	3772.5 ± 209.2 ab	16,027.3 ± 1605.2 ab	784.9 ± 8.9 a	4336.6 ± 78.8 a	26.4 ± 5.8 a	17.9 ± 6.6 a	41.3 ± 2.7 bc
Glyphosate (3.6 g ae L^−1^)	1380.7 ± 12.01 e	11,264.2 ± 1184.4 ab	1672.6 ± 168.8 b	15,860.7 ± 709.2 ab	463.6 ± 39.1 ab	4168.1 ± 139.4 ab	16.9 ± 2.1 ab	15.9 ± 3.6 ab	47.1 ± 4.5 ab
Glyphosate (3.6 g ae L^−1^) + ZnO 25	1805.6 ± 91.6 ab	13,504.7 ± 1190.4 a	4420.1 ± 178.5 a	17,701.1 ± 1597.6 a	467.1 ± 20.7 ab	3847.8 ± 275.9 bc	29.1 ± 9.3 a	18.9 ± 2.4 a	30.1 ± 6.6 c
Glyphosate (17.9 g ae L^−1^)	1442.9 ± 16.2 de	9064.1 ± 699.1 b	1342.5 ± 53.01 b	12,611.3 ± 604.7 c	387.3 ± 11.9 b	3730.4 ± 59.3 c	11.5 ± 0.8 b	6.9 ± 0.6 b	30.6 ± 6.3 c
Glyphosate (17.9 g ae L^−1^) + ZnO 25	1499.9 ± 41.2 cd	11,541.6 ± 632.9 ab	4367.9 ± 100.6 a	14,769.6 ± 864.4 abc	500.4 ± 14.1 ab	3958.4 ± 75.5 abc	16.4 ± 1.6 ab	15.5 ± 1.6 ab	38.7 ± 3.6 bc
CV (%)	-	8.46	-	7.18	-	3.503	25.35	21.73	11.28
*p*-value	0.0071	0.0030	0.1262	0.0018	0.0484	0.0023	1.103	0.0083	0.0001

Values correspond to the mean ± standard deviation. Different letters within the same column indicate significant differences according to the statistical test performed.

## Data Availability

The original contributions presented in this study are included in the article; any additional inquiries can be directed to the corresponding authors.

## Supplementary Materials

The following supporting information can be downloaded at: https://www.mdpi.com/article/10.3390/nano16010039/s1, Table S1: Chemical and physical characteristics of the substrate used for the experiment.

## Abbreviations

ZnO 25	ZnO NPs at 25 mg L^−1^
ROS	Reactive oxygen species
H_2_O_2_	Hydrogen peroxide
MDA	Malondialdehyde
PSI	Photosystem I
PSII	Photosystem II
SPAD	Chlorophyll content index
ZnO NPs	ZnO nanoparticles
ae	acid equivalent
NADP^+^	Nicotinamide Adenine Dinucleotide Phosphate
LHC	Light-harvesting complexes
SOD	Superoxide dismutase
CA	Carbonic anhydrase
