# Supplementary material for: Effect of Zinc Oxide Nanoparticles on the Physiological and Biochemical Responses of Coffea arabica L. Exposed to Glyphosate
Source: Nanomaterials (Basel). 2025 Dec 27;16(1):39. doi: 10.3390/nano16010039 (PMC12788123; doi:10.3390/nano16010039)
Supplement: Supplementary file 1 [file nanomaterials-16-00039-s001.zip › nanomaterials-4055629-supplementary.pdf]

Effect of Zinc Oxide Nanoparticles on the Physiological and Biochemical Responses of *Coffea arabica* L. Exposed to Glyphosate

Leyner Tucto-Vela, Jegnes Benjamín Meléndez-Mori, Eyner Huaman, Amilcar Valle-Lopez and Manuel Oliva Cruz *

Instituto de Investigación para el Desarrollo Sustentable de Ceja de Selva (INDES-CES), Universidad Nacional Toribio Rodríguez de Mendoza (UNTRM), Chachapoyas 01001, Peru
* Correspondence: manuel.oliva@untrm.edu.pe

Supplementary Table S1. Chemical and physical characteristics of the substrate used for the experiment.

Parameter	Unit	Category	Result
pH	pH	–	5.51
Electrical conductivity	(mS/m)	–	211.70
Nitrogen	%	–	7.07
Phosphorus	ppm	–	208.19
Potassium	ppm	–	485.37
Carbon	%	–	9.12
Organic matter	%	–	15.72
Cation exchange capacity	(meq/100g)	–	20.80
	(meq/100g)	Ca^{+2}	11.65
	(meq/100g)	Mg^{+2}	2.53
Exchangeable cations	(meq/100g)	K^{+}	0.80
	(meq/100g)	Na^{+}	0.17
	(meq/100g)	Al^{+3+H+}	0.00

* Data provided by the Laboratorio de Investigación de Suelos y Aguas (LABISAG), Universidad Nacional Toribio Rodríguez de Mendoza de Amazonas (UNTRM). Parameters accredited according to the NTP-ISO/IEC 17025:2017 standard, registration code LE 170 [36].
